# A Case of Auto-Isopathy

**Published:** 1893-07

**Authors:** Med. Buob

**Affiliations:** Fruidenstadt


### A CASE OF AUTO-ISOPATHY.

Translated by A. McNeil, M. D., San Francisco, Cal.

As a sequel of the case reported in the *Allg. Hom. Zeitung*, Nos. 5 and 6 of the present volume, I report this one:

B. K., æt. aged seventy years, an old lady of this place, was treated in the past year by an allopathic physician who diagnosed her case as incipient tuberculosis.

May 15th, 1891, she came under my treatment with symptoms of influenza which was at the time epidemic here. Physical examination showed in the upper lobe of the right lung and in the apex extensive, coarse bronchial *râles;* in the left lung this condition was less clearly marked; respiration weak, in spots bronchial breathing; in both upper lobes amphoric resonance of the respiration. Percussion revealed in both apices anterior dull sounds and in the upper lobes empty ones. Posteriorly in the upper portion tympanitic in places. The febrile movements often exacerbated to 39 and 40 C. (102.2 to 104 F.). Pulse 106 to 112, night-sweats. Cough not very severe, sputa purely purulent, in balls, *sputa criticu-sputa cocta, sputum nummulare.* Stools thin. Rapid sinking of the bodily strength, great emaciation. The treatment was that of Dr. Weihe's method at first, and her condition vacillated between improvement and a standstill until June 6th, when her case threatened to become phthisis florida.

June 6th, I began auto-isopathic treatment, giving her the 100th potency (prepared in the decimal scale up to the 50th and then the centesimal) one drop in 100 *aqua dist.*, a teaspoonful four times a day.

June 7th.—General condition already somewhat better; sleep tolerable, with but few interruptions by cough, fever less, pulse stronger.

June 8th.—Râles decreased; respiration in the upper portion of the lungs, especially the right, more vesicular, dullness not so marked.

June 9th to 12th.—No change.

June 13th.—The patient says that she perceives an actual improvement, and has regained hope of recovery.

June 16th.—Respiration in the apices and upper portions of the lungs regains the vesicular character; dullness is disappearing, although still in the upper posterior region somewhat tympanitic; double sounds (?).

June 16th to 18th.—*Status idem.*

June 19th.—No fever, no night-sweats; expectoration more mucoid; strength increasing; feels well.

June 21st.—Physical examination shows a few dry, fine râles; dullness is gone; percussion sounds correspond closely to the normal. But little cough, expectoration still somewhat mucu-purulent. Auto-Ison.$^{cc}$ (the potentized morbid product from patient) in *aqua dist.*, a teaspoonful three times a day.

I could not send a specimen of her sputa to Dr. Haupt of Chemnitz, specialist in the examination of sputum, in order to discover if bacilla of tuberculosis, as my patient absolutely refused to have it sent. So that there is a possibility that the case may be one of a chronic catarrhal pneumonia, which had become tuberculous.

A few days afterward my patient walked out on sunny days; her strength increased perceptibly, notwithstanding her great age. She feels well and healthy and goes out daily. Since then she has remained healthy and is cheerful.

The most prejudiced skeptic must feel that auto-isopathy is a fact which in the most desperate cases may cause much comfort, and a refusal to test it, even in very far advanced cases, in the face of such facts must be indorsed as inhumane.

In reference to the case of "Fr. Sch." in the *Allg. Hom. Zeitung*, Nos. 5 and 6 of this volume, in which the tubucular bacillus was not discovered, I still believe that if the sputum had been investigated according to the proper mode of conducting bacteriological examinations that the bacillus would have been found, and I, therefore, persist in the view that the case was tuberculous. The examination of the sputum had been conducted by a pharmacist of this city, who is a microscopist in other branches than bacteriology. He had not had the necessary practice, so he himself acknowledged.

Dr. Med. Buob.

Fruidenstadt, Sept., 1892.

